# Optimizing Sperm Cryopreservation from Four Endangered Korean Amphibian Species: Species-Specific Effects of Cryoprotectants and Cooling Regimes on Membrane-Integrity Viability

**DOI:** 10.3390/ani15203013

**Published:** 2025-10-17

**Authors:** Jun-Sung Kim, Da Som Park, Jun-Kyu Park, Ji-Eun Lee, Jeong Chan Moon, Yuno Do

**Affiliations:** 1 Department of Biological Sciences, Kongju National University, Gongju 32588, Republic of Korea; ooo0337@smail.kongju.ac.kr (J.-S.K.); pjk8578@kongju.ac.kr (J.-K.P.); jelee00@smail.kongju.ac.kr (J.-E.L.); 2 Research Center for Endangered Species, National Institute of Ecology, Yeongyang 36531, Republic of Korea; 122dasom@nie.re.kr (D.S.P.); moonjc@nie.re.kr (J.C.M.)

**Keywords:** amphibian, sperm cryopreservation, cryoprotectants, dimethyl sulfoxide, N,N-dimethylformamide, cooling rate, endangered species

## Abstract

**Simple Summary:**

Amphibian populations are rapidly declining, and sperm cryopreservation is essential for conservation. This study tested the post-thaw membrane-integrity viability of sperm from four endangered Korean species following cryopreservation using two different cryoprotectants (dimethyl sulfoxide [DMSO] or N,N-dimethylformamide [DMF] with sucrose) and two cooling methods (freezing at 5 or 10 cm above the liquid nitrogen surface). Low cryoprotectant levels improved membrane-integrity viability, while high concentrations—especially DMSO—were toxic. DMF offered more consistent protection, and slower cooling (10 cm) enhanced viability. Optimal conditions were 15% DMSO at 10 cm for *Dryophytes suweonensis* (86.5%), 10% DMF at 10 cm for *Pelophylax chosenicus* (75.5%), and 10% DMSO at 5 cm for *Kaloula borealis* (81.6%). *Hynobius yangi* showed low membrane-integrity viability overall, peaking at 19.7% with 15% DMF at 5 cm.

**Abstract:**

Global amphibian populations are declining rapidly and the development of effective cryopreservation protocols for germ cells has become a critical tool in ex situ conservation programs. Post-thaw membrane-integrity viability in four endangered Korean amphibians (*Dryophytes suweonensis*, *Pelophylax chosenicus*, *Kaloula borealis*, and *Hynobius yangi*) were evaluated. Sperm were cryopreserved using dimethyl sulfoxide (DMSO) or N,N-dimethylformamide (DMF) at 10–30% (*v*/*v*) in combination with 0.6 M sucrose, and were frozen at two suspension heights (5 cm vs. 10 cm) above liquid nitrogen. Post-thaw membrane-integrity viability was assessed using a SYBR-14/propidium iodide membrane-integrity assay (LIVE/DEAD kit). Low concentrations of permeating cryoprotectants (CPs) improved membrane-integrity viability, whereas high concentrations led to high toxicity, particularly with DMSO. Across species, DMF produced the highest membrane-integrity viability and the most consistent performance. The cooling rate influenced membrane-integrity viability, with samples frozen at 10 cm exhibiting greater viability, reflecting the balance between intracellular ice formation during rapid cooling and solution effects during slow cooling. Optimal conditions for *D. suweonensis* were 15% DMSO at 10 cm (86.5% membrane-integrity viability); for *P. chosenicus,* 10% DMF at 10 cm (75.5%); and for *K. borealis*, 10% DMSO at 5 cm (81.6% membrane-integrity viability). *Hynobius yangi* showed modest improvement under 15% DMF at 5 cm (19.7%), although overall membrane-integrity viability was low. ED_50_ modeling indicated species-specific thresholds requiring low CP concentrations. Sperm cryopreservation outcomes in amphibians are strongly influenced by CP type, concentration, cooling regime, and species physiology. GLM and ED_50_ modeling provide a methodological framework for refining cryopreservation strategies for non-model, endangered species.

## 1. Introduction

Global amphibian populations have declined at a faster rate than that of other vertebrate groups in recent decades, which is considered a prime example of the ongoing biodiversity crisis. According to the 2023 IUCN Global Amphibian Assessment, approximately 41% of all amphibian species are rated as threatened (critically endangered, endangered, or vulnerable), and approximately 60% of salamanders fall within this threat category [1]. This decline is caused by complex factors (habitat loss and fragmentation, changes in landscape, deterioration of water quality due to agricultural/urbanization and road opening, rainfall–water temperature–predation period fluctuations due to climate change, and infectious diseases such as chytrid fungi) rather than by a single cause [2,3]. A global meta-analysis indicated that chytrid fungi have caused a rapid population collapse in hundreds of species over the past five decades [4].

Amphibians are vulnerable to environmental changes because of their physiological characteristics, which depend on skin breathing and highly permeable skin, and their biological life cycles, which go through both aquatic and terrestrial stages [5,6]. Disturbances that occur at any stage of life threaten the sustainability of a population, which requires a strategy that combines in situ and ex situ conservation [7,8]. Within the One Conservation framework, these actions are treated as a single, connected system linking field populations, conservation-breeding programs, and knowledge transfer. In this context, genetic resource banking (biobanking) and allied reproductive biotechnologies—such as cryopreservation of gametes and artificial insemination—serve as connectors that enable assisted gene flow and the maintenance of allelic diversity and demographic resilience across fragmented populations [9,10].

Sperm cryopreservation is well suited to amphibian conservation because sperm are easier to obtain than oocytes, require less invasive handling, and can be collected in larger quantities per individual [11,12]. After thawing, cryopreserved sperm can be used with assisted reproductive technologies—including in vitro fertilization (IVF) and intracytoplasmic sperm injection (ICSI)—to restore fertilization and development in at-risk populations [11,13], with recent work in *Xenopus laevis* and urodeles demonstrating functional developmental outcomes from assisted reproduction and cryopreserved gametes [14]. Beyond storage, sperm banking and allied assisted reproductive technologies (ART) serve as population-management tools by enabling assisted gene flow, reducing inbreeding and bottlenecks, and linking ex situ and in situ populations within integrated conservation programs [15].

The protocols adopted for each of the model species specify the physical and chemical variables. For example, in *X. laevis*, motility inhibiting saline + 5% dimethyl sulfoxide (DMSO) + sucrose, and freezing and room-temperature thawing (approximately 40 s) in a vapor layer approximately 10 cm above the surface of liquid nitrogen, have been reported as optimal, showing the effect of cooling pathways and microgeometry (sample volume and shape) on performance [16,17]. This case serves as the basis for adopting freezing height as an operational variable in this study.

The success or failure of cryopreservation depends on the cooling rate. The classical two-factor hypothesis explains that excessively slow cooling is a solution effect (high concentration of solute exposure and dehydration stress), and excessively fast fatal damage is caused by intracellular ice formation [16,18]. Experimental and theoretical accumulations support the existence of this optimal cooling rate window, and in actual operation, the sample volume, container, and steam bed height indirectly control the cooling rate [17].

The existing literature focuses on a narrow set of model species, and heterogeneity in cryoprotectant type, concentration, and cooling conditions across laboratories hampers comparison and standardization [16,19]. Because samples from endangered species are limited, studies must extract maximal information from minimal material through rigorous design and analysis. This study evaluated four amphibians from the Korean Peninsula (*Dryophytes suweonensis*, *Pelophylax chosenicus*, *Kaloula borealis*, *Hynobius yangi*) across combinations of cryoprotectant type, cryoprotectant concentration, and vapor-phase cooling height above liquid nitrogen. The objective was to quantify species-specific effects and interactions on post-thaw membrane-integrity viability and to summarize concentration–response sensitivity using a predefined analysis plan. The physical cooling context was incorporated by relating vapor-phase positions to characteristic cooling rates, providing a link to parameter-adjustable operating protocols [20].

Four species were evaluated using the same analysis pipeline to obtain the interspecies response spectra. The study proves the interaction of CP × concentration × prefreeze height and provides predictability through ambient prediction and dose–response curves. The analysis results were reduced to the recommended conditions for each type and are presented as customized freeze preservation guidelines that can be applied immediately by conservation institutions, breeding centers, and genetic resource banks. In this process, the standardization and generalization of frozen sperm preservation in amphibian conservation biology are taken a step further by linking the principles established in the model species (the two-factor hypothesis and the physical meaning of vapor layer freezing) and the biobanking framework (maintenance of genetic diversity and mating management) required in international conservation practices with empirical data.

## 2. Materials and Methods

### 2.1. Study Population

All animals were collected from the wild. In early April 2025, sexually mature adult males of *H. yangi* were collected in Busan Metropolitan City, Republic of Korea. From July to August 2025, adult males of three anuran species were collected at the following localities: *D. suweonensis* (Buyeo-gun), *P. chosenicus* (Cheongyang-gun), and *K. borealis* (Daejeon Metropolitan City). Animals were captured by hand. Reproductively active males were identified by nuptial pads in anurans and by a swollen, prominent cloaca in *H. yangi*. Collections of endangered species were authorized by the Ministry of Environment of the Republic of Korea (permit nos. EB202503ECP0016 for *H. yangi*; ED202505ECP0008 for *D. suweonensis*, *P. chosenicus*, and *K. borealis*). All procedures were approved by the Experimental Animal Ethics Committee of Kongju National University (KNU_2025-01).

### 2.2. Sperm Collection

We used 15 adult males for *D. suweonensis*, 15 for *P. chosenicus*, 15 for *K. borealis*, and 5 for *H. yangi* as biological replicates. The animals were maintained in dechlorinated freshwater in collection containers (22 × 14 × 16 cm) for 30 min for acclimation and hydration prior to handling. An Amphibian Ringer’s solution was prepared by dissolving 6.6 g NaCl, 0.15 g KCl, 0.15 g CaCl_2_, and 0.2 g NaHCO_3_ in 1 L of distilled water. For anesthesia, a 0.1% MS-222 solution was prepared by dissolving 1 g of ethyl 3-aminobenzoate methanesulfonate (E10521; Sigma-Aldrich, St. Louis, MO, USA) and 1 g of sodium bicarbonate in 1 L of distilled water. For hormonal induction of spermiation, human chorionic gonadotropin (hCG; C1063; Sigma-Aldrich, St. Louis, MO, USA) or luteinizing hormone-releasing hormone analog (LHRHa; L4513; Sigma-Aldrich, St. Louis, MO, USA) was dissolved in Amphibian Ringer’s solution. Adult males of *D. suweonensis*, *H. yangi*, and *K. borealis* received intraperitoneal injections of 200 μL hCG (300 IU) per animal, whereas *P. chosenicus* males received 200 μL LHRHa at 0.5 mg g^−1^ body weight. All injections were administered using a 1 mL, 31-gauge insulin syringe. Intraperitoneal injections were performed in conscious animals to avoid potential interference of immersion anesthesia with endocrine induction and to minimize anesthetic exposure in wild individuals. To limit distress, injections were delivered by trained personnel under gentle manual restraint using 31-gauge needles, with ≤5 s needle dwell time and a lower-right abdominal quadrant entry. Following hormone administration, animals were anesthetized by partial immersion of the abdomen in 0.1% MS-222 for up to 20 min, after which gentle abdominal pressure was applied to express sperm via the cloaca. White seminal fluid or urine containing sperm was collected into sterile 1.5 mL Eppendorf tubes (MCT-150-C; Corning, Corning, NY, USA), immediately diluted 1:2 (*v*/*v*) with Amphibian Ringer’s solution as an isotonic extender, and held on ice (0–4 °C) in the dark for up to 5 h prior to cryopreservation. Following collection, the individuals were rehydrated in dechlorinated freshwater for 1 h, allowed to recover until normal righting, and blinking reflexes were observed, and then released at the original capture site. Immediately prior to cryopreservation, the initially diluted sperm was further diluted on ice with Amphibian Ringer’s solution to a final concentration of 1.4–1.5 × 10^6^ cells mL^−1^, determined with a disposable hemocytometer (I5.N015; INCYTO, Cheonan, Republic of Korea).

Following this final dilution, baseline membrane-integrity viability was assessed in the dark using a LIVE/DEAD Sperm Viability Kit (L7011; Thermo Fisher Scientific, Waltham, MA, USA). The SYBR-14 stock solution was diluted 1:50 (*v*/*v*) in Amphibian Ringer’s solution, and undiluted propidium iodide (2.4 mM) was used. For staining, 5 μL of the sperm suspension was mixed with 4 μL of diluted SYBR-14 and incubated for 5 min, followed by 1 μL of propidium iodide and a further 1 min. A 10 μL aliquot was loaded onto a disposable hemocytometer and examined under fluorescence microscopy at ×200. Cells with green fluorescence (SYBR-14 positive) were counted as viable; those with red fluorescence (propidium iodide positive) were counted as non-viable (Table 1). In this study, “membrane-integrity viability” refers to membrane-integrity-based viability measured with the SYBR-14/propidium iodide LIVE/DEAD assay. Functional endpoints—including total and progressive motility, morphology, and fertilizing capacity—were not assessed in this dataset.

### 2.3. Cryoprotectant Preparation

Cryoprotectant (CP) solutions were prepared by dissolving 20.538 g of sucrose in Amphibian Ringer’s solution to which dimethyl sulfoxide (DMSO; D8418; Sigma-Aldrich, St. Louis, MO, USA) or N,N-dimethylformamide (DMF; 72438; Sigma-Aldrich, St. Louis, MO, USA) was added to achieve the desired concentration. The final volume was adjusted to 100 mL using Amphibian Ringer’s solution and the prepared solutions were stored at 4 °C until use. Six CP formulations were tested, comprising DMSO at 10%, 20%, and 30% (*v*/*v*), each combined with 0.6 M sucrose, and DMF at 10%, 20%, and 30% (*v*/*v*), each combined with 0.6 M sucrose.

### 2.4. Sperm Cryopreservation

Freshly collected sperm (≤5 h post-collection) were mixed 1:1 (*v*/*v*) on ice with cryoprotectant (CP) stock solutions pre-chilled to 4 °C (10, 20, or 30% [*v*/*v*] DMSO or DMF in 0.6 M sucrose), yielding final CP concentrations of 5, 10, or 15% (*v*/*v*) and a final sucrose concentration of 0.3 M. Aliquots of 50 μL were loaded into 0.25 mL plastic straws (005565; IMV Technologies, L’Aigle, France) and sealed with poly(vinyl alcohol) (341584; Sigma-Aldrich, St. Louis, MO, USA). Straws were equilibrated at 4 °C for 10 min prior to freezing. For cooling, straws were suspended 5 cm or 10 cm above liquid nitrogen for 10 min inside a polystyrene container to achieve vapor-phase freezing; under similar experimental conditions these positions correspond to approximate cooling rates of −32 to −45 °C·min^−1^ (5 cm) and −20 to −29 °C·min^−1^ (10 cm) [21]. Straws were then plunged into liquid nitrogen and stored until use.

Final CP concentrations of 5%, 10%, and 15% were selected to cover the low-concentration window repeatedly reported as effective in amphibians, while avoiding toxicity often observed at higher levels, particularly ≥20% DMSO [12,16,19]. Suspension heights of 5 cm and 10 cm were chosen to bracket two vapor-phase regimes characterized previously [21]. We evaluated 12 treatment combinations per species (2 cryoprotectants × 3 final CP concentrations × 2 suspension heights). For *D. suweonensis* and *P. chosenicus*, we prepared seven 50 µL straws per condition. For *K. borealis* and *H. yangi*, we prepared five 50 µL straws per condition. This yielded the following numbers of post-thaw technical replicates per species—*D. suweonensis N* = 84 (12 × 7), *P. chosenicus N* = 84 (12 × 7), *K. borealis N* = 60 (12 × 5), and *H. yangi N* = 60 (12 × 5). Technical replicates were defined at the straw level and biological replicates at the individual-male level. Parameter ranges followed amphibian cryopreservation literature supporting low-range CP concentrations and vapor-phase freezing at 5–10 cm above liquid nitrogen [12,17,20].

### 2.5. Sperm Thawing and Viability Assessment

After at least one week of storage, the straws were retrieved from liquid nitrogen and thawed by immersion in a 40 °C water bath for 5 s. Immediately thereafter, the straws were placed on ice to prevent temperature rise. The thawed sperm–CP mixture was transferred to sterile Eppendorf tubes and gently mixed by pipetting. Post-thaw membrane-integrity viability was evaluated using the same method as for the initial membrane-integrity viability assessment, with a LIVE/DEAD membrane-integrity viability Kit (L7011; Thermo Fisher Scientific, Waltham, MA, USA).

### 2.6. Statistical Analysis

All statistical analyses were conducted in R (version 4.3.2) in a 64-bit Linux environment using tidyverse (1.3.2), binom (1.1-1), broom (1.0.5), emmeans (1.8.8), lme4 (1.1-34), glmmTMB (1.1.7), geepack (1.3-9), sandwich (3.0-2), and lmtest (0.9-40). Condition- or species-level means are reported as mean ± standard error (SE) across straws (technical replicates). Confidence intervals of 95% (95% CI) are Wilson intervals computed from aggregated counts (alive/total). Within-species comparisons among conditions used binomial models or Fisher’s exact tests, with multiple-comparison control by Benjamini–Hochberg FDR (α = 0.05). For Fisher’s tests, each condition was compared to the species-specific baseline, and when an individual contributed to multiple conditions, counts were treated independently at the condition level; individual clustering was assessed in robustness analyses. For each experimental replicate, counts of viable (SYBR-14 positive) and non-viable (propidium iodide positive) spermatozoa were recorded, and membrane-integrity viability was calculated as follows:

(1) $$membrane-integrity\;viability(v)=\frac{viable(alive)}{viable(alive)+non-viable(dead)}$$

In model descriptions, we refer to the binomial response as “survival probability” (i.e., the model-based probability that a cell is viable) to distinguish the statistical parameter from the biological measurement of membrane-integrity viability.

Species identity was treated as a fixed factor in a predefined order (*D. suweonensis* > *P. chosenicus* > *K. borealis* > *H. yangi*). We encoded species using treatment contrasts with *D. suweonensis* as the reference level; thus, the coefficients β_species,i quantify log-odds differences relative to *D. suweonensis*. In models including interactions, species main effects are interpreted at the reference levels of the interacting factors. The experimental conditions were expressed as combinations of cryoprotectant (CP) type, CP concentration, and pre-freezing height. The CP concentration (in % *v*/*v*) and pre-freezing height (in cm) were converted to numeric values and standardized (*z*-scores) before modeling using Equation (2):

(2) $$z(x)=\frac{x-\bar{x}}{SD(x)}$$

where $\bar{x}$ and SD(*x*) are the mean and standard deviation across all observations, respectively.

For each species, a baseline condition was defined by prioritizing treatments explicitly labeled as “control,” “no cryoprotectant,” “untreated,” or “baseline.” If no such labels were present, the condition with the largest sample size was used. Pairwise differences in membrane-integrity viability between each treatment and the species-specific baseline were evaluated using Fisher’s exact test applied to 2 × 2 contingency tables of live and dead counts. Odds ratios (OR) and corresponding *p*-values were computed. Multiple comparisons were adjusted using the Benjamini–Hochberg false discovery rate (FDR) procedure, with a significance threshold of α = 0.05 applied after FDR correction. If the same individual was measured across multiple conditions, counts were treated independently at the condition level for Fisher’s test, whereas individual clustering was addressed in the robustness analyses described below.

A binomial logistic regression model (GLM) with a logit link was fitted to assess the combined effects of the predictors.

(3) $$logit\left(p_{ijkl}\right)=\beta_{0}+\beta_{{species}_{i}}+\beta_{{CP}_{j}}+\beta_{2}z\left({height}_{l}\right)+\beta_{3}({CP}_{j}\times z\left({conc}_{k}\right))$$

where *p*_*ijkl*_ is the survival probability (model-estimated probability of viability); $\beta_{{species}_{i}}$ and $\beta_{{CP}_{j}}$ are categorical effects; and the interaction term (CP_*j*_ × *z*(conc_*k*_)) accounts for CP-specific differences in concentration–response slopes. Parameter estimates were reported with Wald 95% confidence intervals. Overdispersion was assessed as Pearson’s χ^2^ statistic divided by the residual degrees of freedom.

Marginal predictions for each species–CP combination were obtained using the emmeans package (1.11.2), setting all standardized covariates to zero (i.e., average experimental conditions). Dose–response relationships were analyzed by fitting a species-CP-specific binomial GLM with concentration as the sole predictor:

(4) $$logit\left(p\right)=\alpha +\beta \cdot conc$$

From which the median effective concentration (ED_50_) was calculated as −α/β. Standard errors and 95% confidence intervals (CIs) for ED_50_ were estimated using the delta method. Only pairs of CP species with at least three distinct concentrations were included in the ED_50_ estimation.

The robustness of the primary GLM results was evaluated using four complementary modeling approaches designed to address the potential non-independence of observations and overdispersion. Generalized linear mixed models (GLMM; lme4::glmer) with a binomial logit link were fitted by incorporating a random intercept for individual identity nested within species to account for repeated measurements of the same individuals. Beta-binomial GLMMs (glmmTMB) were applied using the same fixed and random effects structure, but with a beta-binomial error distribution to explicitly model the overdispersion. Generalized estimating equations (GEE; geepack::geeglm) with a binomial logit link and an exchangeable correlation structure were used, specifying individual identity as the clustering variable. A cluster-robust GLM was fitted (sandwich:vcovCL combined with lmtest:coeftest) by applying robust standard errors clustered by individual identities to a standard binomial GLM framework. These additional models facilitated the assessment of whether the direction and significance of the key predictors were consistent across different correlation structures and distributional assumptions.

## 3. Results

### 3.1. Post-Thaw Membrane-Integrity Viability Across Species and Treatments

**Figure 1:**
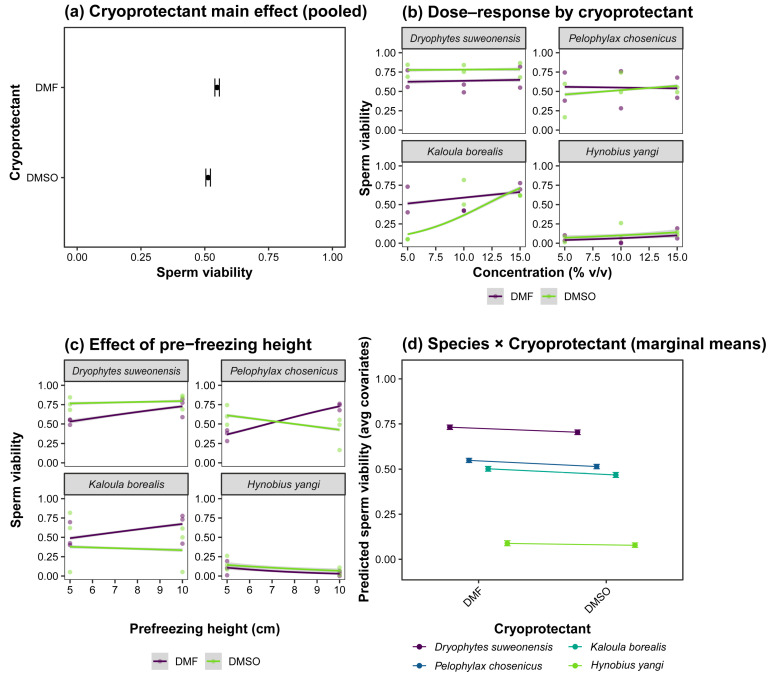
Post-thaw membrane-integrity viability across species and treatments. (**a**) Mean membrane-integrity viability of *Dryophytes suweonensis*, *Pelophylax chosenicus*, *Kaloula borealis*, and *Hynobius yangi* across cryoprotectants. (**b**) Dose–response patterns showing stable membrane-integrity viability at 10% dimethyl sulfoxide (DMSO) but sharp decline at 30%, and highest membrane-integrity viability at 10% N,N-dimethylformamide (DMF), with gradual decline at higher concentrations. (**c**) Effect of pre-freezing suspension height (5 vs. 10 cm above liquid nitrogen) on membrane-integrity viability, reflecting cooling rate differences. (**d**) Model-based marginal predictions indicating 10% DMF + sucrose as the most favorable formulation, particularly for *K. borealis* and *P. chosenicus*, while 30% formulations consistently underperformed.

Overall, post-thaw membrane-integrity viability differed markedly among the four focal species, *D. suweonensis*, *P. chosenicus*, *K. borealis*, and *H. yangi* (Table 2 and Figure 1a). Across all treatments, *D. suweonensis* exhibited the highest mean membrane-integrity viability, whereas *H. yangi* showed consistently low values. *P. chosenicus* and *K. borealis* were intermediate overall, with clear improvements under specific cryoprotectant–concentration–height combinations. The condition-specific patterns were consistent with the dose–response signature (Figure 1b). With DMSO, membrane-integrity viability was relatively stable at 10% (*v*/*v*), with 0.6 M sucrose, but declined sharply at 30% DMSO. With DMF, the highest viability was observed at 10% concentration, which decreased gradually as the concentration increased.

These patterns concur with the species-level summaries in Table 3. The physical pre-freezing context showed additional effects (Figure 1c). Variations in suspension height above liquid nitrogen were associated with measurable differences in post-thaw membrane-integrity viability within several species–CP combinations, indicating that the cooling regime contributes to overall performance beyond CP type and concentration. Model-based marginal means further clarified the species–CP contrasts (Figure 1d). The predicted membrane-integrity viability, with covariates standardized to their means, identified 10% DMF with sucrose as the most consistently favorable formulation across species, particularly for *K. borealis* and *P. chosenicus*, whereas 30% CP formulations underperformed.

**Figure 2:**
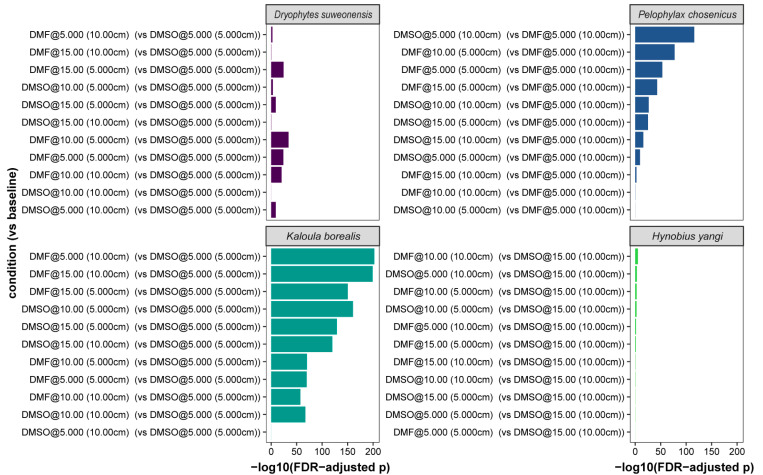
Condition-specific significance of post-thaw membrane-integrity viability relative to species-specific baselines, shown as −log10 (FDR-adjusted *p*) from Fisher’s exact tests. “@” denotes the final cryoprotectant concentration; parentheses denote the pre-freezing height (cm). Abbreviations: DMSO, dimethyl sulfoxide; DMF, N,N-dimethylformamide. For GLM-based comparisons reported elsewhere, the reference species is *D. suweonensis*, and coefficients for other species are interpreted relative to this reference.

The species-specific optima were as follows. *Dryophytes suweonensis* achieved the highest post-thaw membrane-integrity viability with 15% DMSO at 10 cm (86.5%, 95% CI 83.3–89.3). *Pelophylax chosenicus* performed best with 10% DMF at 10 cm (76.1%, 95% CI 73.1–78.9). *Kaloula borealis* reached its maximum with 10% DMSO at 5 cm (81.6%, 95% CI 77.3–85.5). *Hynobius yangi* showed low values overall, but the highest outcome occurred with 15% DMF at 5 cm (19.7%, 95% CI 13.5–26.7). Condition-wise improvements relative to species-specific baselines were supported by Fisher’s exact tests with FDR adjustment (Table 3 and Figure 2).

### 3.2. Effects of Species, Cryoprotectant, and Cooling Conditions on Post-Thaw Membrane-Integrity Viability

**Figure 3:**
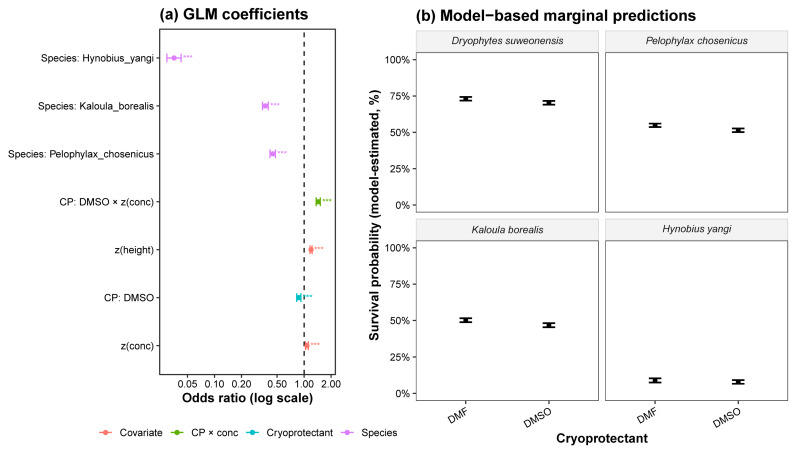
Generalized linear model (GLM) results for post-thaw membrane-integrity viability. (**a**) Forest plot of GLM coefficients expressed as odds ratios (95% CI) on a log scale. Predictors include standardized covariates (*z*-transformed concentration and freezing height), cryoprotectant (CP: DMSO vs. DMF, both supplemented with 0.6 M sucrose), and species effects. Asterisks indicate significance levels (*** *p* < 0.001). (**b**) Model-based marginal predictions of survival probability (model-estimated probability of viability) across species and cryoprotectant types (all with 0.6 M sucrose), estimated at average experimental conditions (*z*(concentration) = *z*(height) = 0). Error bars represent 95% confidence intervals.

Generalized linear modeling (GLM, binomial logit link) indicated marked effects of species, cryoprotectant (CP) type, concentration (*z*-scored), and suspension height above liquid nitrogen (*z*-scored) on post-thaw membrane-integrity viability (Figure 3a). Species differences were pronounced, with *K. borealis* exhibiting the highest overall membrane-integrity viability and *H. yangi* consistently showing the lowest. Cryoprotectant concentration had a strong negative effect (*z*(conc)), with higher concentrations reducing membrane-integrity viability. The interaction between DMSO and the concentration (CP × *z*(conc)) was also negative, indicating a steep decline in membrane-integrity viability at high DMSO concentrations, whereas DMF showed a more gradual, concentration-dependent reduction. Suspension height was also statistically significant, with samples positioned higher above the liquid nitrogen (10 cm), showing modestly improved membrane-integrity viability compared to samples positioned lower above the liquid nitrogen (5 cm).

Model-based marginal predictions clarified species–CP contrasts under average conditions (*z*(conc) = *z*(height) = 0) (Figure 3b and Table 4). For *D. suweonensis*, membrane-integrity viability was the highest with DMF (73.2%, 95% CI: 71.9–74.4%) and DMSO (70.4%, 95% CI: 69.1–71.7%). *Pelophylax chosenicus* showed intermediate membrane-integrity viability with DMF at 54.9% (95% CI: 53.7–56.0%) and DMSO at 51.5% (95% CI: 50.2–52.7%). *Kaloula borealis* maintained relatively high and stable membrane-integrity viability, with DMF (50.2%, 95% CI: 48.9–51.5%) outperforming DMSO (46.8%, 95% CI: 45.4–48.2%). In contrast, *H. yangi* showed consistently low values with DMF (8.8%, 95% CI: 7.5–10.3%) and DMSO (7.8%, 95% CI: 6.6–9.1%), both below 10%.

### 3.3. Inter-Specific Variation and Optimal Conditions

Post-thaw membrane-integrity viability differed markedly among species. The highest single-condition outcome was observed in *D. suweonensis* with 15% DMSO at 10 cm (86.5%, 95% CI 83.3–89.3). *Pelophylax chosenicus* performed best with 10% DMF at 10 cm (75.5%, 95% CI 73.1–78.9). *Kaloula borealis* reached its maximum with 10% DMSO at 5 cm (81.6%, 95% CI 77.3–85.5). *Hynobius yangi* showed low values overall, but the highest outcome occurred with 15% DMF at 5 cm (19.7%, 95% CI 13.5–26.7). These per-species optima are summarized in Table 5, and condition-wise improvements relative to species-specific baselines are supported by Fisher’s exact tests with FDR adjustment (Table 3 and Figure 2).

Although formal modeling captured the main effects of species, cryoprotectant type, and cooling height on the endpoint, dose–response analyses (ED50 estimates) refined concentration-specific thresholds. Pronounced concentration dependence was evident in *K. borealis* and *P. chosenicus* (e.g., ED50 ≈ 4.1% DMF [95% CI 2.5–5.8] and 8.6% DMSO [95% CI 7.2–10.0], respectively), indicating that maximal effectiveness occurred at relatively low concentrations. In *D. suweonensis*, ED50 estimates were unstable or negative, suggesting inconsistent concentration effects, whereas in *H. yangi*, high ED50 values indicated limited improvement with increasing concentration.

Across datasets, low-range CP concentrations (5–15% *v*/*v*) with 0.6 M sucrose and vapor-phase cooling at 5–10 cm performed best overall. A robust option across species was 10% DMF at 10 cm—particularly for *P. chosenicus*—whereas *K. borealis* achieved its highest outcome with 10% DMSO at 5 cm. *H. yangi* exhibited only marginal gains under both DMF and DMSO regimens, underscoring inherent species-specific limitations in cryopreservation success (Table 5).

## 4. Discussion

The membrane-integrity viability pattern observed in this study indicated that the interaction between the concentration of the permeating cryoprotectant (permeating CP) and cooling rate was the decisive factor. Both DMSO and DMF showed protective effects at low concentrations; however, their toxicity increased rapidly at high concentrations. This “low-concentration advantage—high-concentration inferiority” pattern has been reported in a variety of species, including fish, amphibians, and mammals [22,23,24]. DMSO is characterized by a rapid conversion to toxicity as the concentration increased, and in the GLM analysis of the current study, both the concentration main effect and the DMSO × concentration interaction term were estimated to be negative, which is consistent with previous reports. In contrast, DMF showed a relatively weak concentration dependence, which can be interpreted as being due to properties that are different to DMSO in terms of membrane permeability, hydration response, and osmotic balance [12,25].

It is highly likely that sucrose, which was uniformly added under all conditions in this study, also played an important role. Non-permeating saccharides increase extracellular osmosis to prevent excessive retention of moisture in the cell and inhibit crystal nucleation by reinforcing the solute effect during the freeze–thaw process. Reports that sucrose supplementation increases the protective effect of CP in *Rana* frog sperm cryopreservation studies [12,26,27] support the maintenance of a relatively high membrane-integrity viability, even under low-concentration CP. Sucrose may act as an auxiliary agent that delays CP toxicity and maximizes efficiency under low-concentration conditions. The effect of the cooling rate was evident. Positioning straws 5 versus 10 cm above liquid nitrogen modulated the cooling rate, and the higher membrane-integrity viability at 10 cm indicates sensitivity to the window between intracellular ice formation during overly rapid cooling and solution effects during overly slow cooling [21,28].

Differences between species have been reported. *Kaloula borealis* showed a consistently high membrane-integrity viability, whereas *H. yangi* showed a low overall membrane-integrity viability. This pattern is consistent with previous reports [23,29] in which membrane lipid composition and sperm morphological characteristics affected CP sensitivity. For example, membrane unsaturation and the cholesterol ratio are known to be key factors determining CP permeability and osmotic stress resistance. Sperm morphological factors, such as head size and surface area-to-volume ratio, also affect CP penetration kinetics and water transfer rate. The distinctly different response curves of species in the current study can be interpreted as a phenotype of this membrane as well as morphological and metabolic heterogeneity [30,31].

The low post-thaw membrane-integrity viability of *H. yangi* cannot be explained simply by differences in the types of cryoprotectants or cooling conditions. The low membrane-integrity viability observed in this species is likely to be due to the physiological properties of the species itself, such as cell membrane composition, metabolic sensitivity of spermatozoa, and the ability to regulate moisture. Castro et al. [32] and Mandal et al. [33] reported that membrane lipid composition and sperm morphology were important factors determining cryosensitivity; this is supported by the current study. Similar patterns have been reported for other amphibian species. In Fowler’s toad (*Anaxyrus fowleri*), distinct differences in fertility and embryo incidence have been observed depending on the CP and cooling rate conditions [21]. In Eastern Dwarf tree frogs (*Litoria fallax*), cases of development from stored sperm to tadpoles and adults have been reported, but the rates are very low [34]. These cases show that cryopreservation efficiency can be extremely low in some species, which concurs with the low membrane-integrity viability observed in *H. yangi* in this study.

The low membrane-integrity viability of *H. yangi* may be difficult to improve by simply adjusting the existing CP combinations or cooling conditions, and the introduction of adjuvants that increase membrane stability or mitigate reactive oxygen species accumulation may be necessary. Antioxidants, such as melatonin and uric acid, substantially improve membrane-integrity viability in amphibian sperm cryopreservation studies [35], suggesting the possibility of new adjuvant strategies in species such as *H. yangi*, which exhibit low post-thaw membrane-integrity viability.

The GLM and ED_50_-based models applied in the current study illustrate how different statistical approaches can be used in a complementary manner. The GLM was useful for estimating the major effects of CP concentration and cooling rate under multivariate conditions, whereas ED_50_ estimation quantified concentration–response sensitivity. Incorporating both frameworks into future cryopreservation research may provide a more comprehensive understanding of treatment effects.

## 5. Conclusions

This study proposes a dual-model framework for amphibian sperm cryopreservation that optimizes species-specific protocols and integrates evidence across species, conditions, and technical environments. Standardizing on a membrane-integrity viability endpoint enabled field-feasible cross-species comparisons. A comprehensive appraisal of cryopreservation performance requires multi-layered analyses that go beyond simple viability. Future work should incorporate segmented cryoprotectant concentration designs, precise control and transparent reporting of cooling rates, and functional endpoints such as CASA-based total and progressive motility, morphology, and fertilization or embryonic development outcomes. Extending the present framework in this direction will broaden its utility for mechanistic inference and will strengthen experimental reproducibility across taxa and settings.

## Figures and Tables

**Table 1:** Baseline (pre-freeze) sperm membrane-integrity viability (mean ± SE, %; 95% CI) by species.

Species	Alive/Total	Mean Viability (%) ± SE (%)	95% CI (%)
*Dryophytes suweonensis*	99/102	97.0% ± 1.7%	91.7–99.0%
*Pelophylax chosenicus*	330/343	96.2% ± 1.0%	93.6–97.8%
*Kaloula borealis*	270/282	95.7% ± 1.2%	92.7–97.5%
*Hynobius yangi*	252/263	95.8% ± 1.2%	92.7–97.6%

*n* (individuals at baseline)—*D. suweonensis* 15, *P. chosenicus* 15, *K. borealis* 15, *H. yangi* 5. Alive/Total are aggregated cell counts across all individuals. SE is the binomial standard error from Alive/Total. 95% CI is the Wilson interval.

**Table 2:** Overall post-thaw membrane-integrity viability (mean ± SE, %; 95% CI) across species.

Species	Alive/Total	Mean Viability (%) ± SE (%)	95% CI (%)
*Dryophytes suweonensis*	4337/6103	71.1% ± 0.6%	69.9–72.2%
*Pelophylax chosenicus*	5157/9656	53.4% ± 0.5%	52.4–54.4%
*Kaloula borealis*	3333/7030	47.4% ± 0.6%	46.2–48.6%
*Hynobius yangi*	143/1633	8.8% ± 0.7%	7.5–10.2%

*n* (biological replicates, males)—*D. suweonensis* 15, *P. chosenicus* 15, *K. borealis* 15, *H. yangi* 5. *N* (post-thaw technical replicates, straws)—*D. suweonensis* 84, *P. chosenicus* 84, *K. borealis* 60, *H. yangi* 60. Alive/Total are aggregated cell counts across all post-thaw straws and conditions. SE is the binomial standard error from Alive/Total. CI of 95% is the Wilson interval.

**Table 3:** Condition-specific Fisher’s exact test results versus baseline condition for each species (proportions with 95% CI; odds ratio with 95% CI; BH–FDR-adjusted *p*-values).

Species	Condition	vs_Baseline	Condition Mean (%, 95% CI)	Baseline Mean (%, 95% CI)	Odds Ratio [95% CI]	*p*-Value	FDR-Adjusted *p*	Significance
*Dryophytes* *suweonensis*	DMF@5.000 (10.00 cm)	DMSO@5.000 (5.000 cm)	77.4 (73.6 ± 0.7)	84.4 (81.2–87.2)	0.63 [0.46–0.86]	0.003	0.004	**
	DMF@15.00 (10.00 cm)	DMSO@5.000 (5.000 cm)	81.9 (78.4 ± 4.9)	84.4 (81.2–87.2)	0.83 [0.60–1.16]	0.264	0.331	ns
	DMSO@15.00 (10.00 cm)	DMSO@5.000 (5.000 cm)	86.5 (83.3 ± 9.3)	84.4 (81.2–87.2)	1.19 [0.83–1.70]	0.341	0.417	ns
	DMSO@10.00 (10.00 cm)	DMSO@5.000 (5.000 cm)	84.1 (80.8 ± 6.9)	84.4 (81.2–87.2)	0.98 [0.70–1.36]	0.935	0.979	ns
	DMF@10.00 (10.00 cm)	DMSO@5.000 (5.000 cm)	58.8 (54.5 ± 3.0)	84.4 (81.2–87.2)	0.26 [0.20–0.35]	<0.001	<0.001	***
	DMF@10.00 (5.000 cm)	DMSO@5.000 (5.000 cm)	48.8 (44.3 ± 3.4)	84.4 (81.2–87.2)	0.18 [0.13–0.24]	<0.001	<0.001	***
	DMF@15.00 (5.000 cm)	DMSO@5.000 (5.000 cm)	54.9 (50.2 ± 9.4)	84.4 (81.2–87.2)	0.22 [0.17–0.30]	<0.001	<0.001	***
	DMF@5.000 (5.000 cm)	DMSO@5.000 (5.000 cm)	55.7 (51.2 ± 0.1)	84.4 (81.2–87.2)	0.23 [0.17–0.31]	<0.001	<0.001	***
	DMSO@10.00 (5.000 cm)	DMSO@5.000 (5.000 cm)	75.4 (71.3 ± 9.1)	84.4 (81.2–87.2)	0.56 [0.41–0.78]	<0.001	<0.001	***
	DMSO@15.00 (5.000 cm)	DMSO@5.000 (5.000 cm)	68.2 (63.9 ± 2.2)	84.4 (81.2–87.2)	0.40 [0.29–0.54]	<0.001	<0.001	***
	DMSO@5.000 (10.00 cm)	DMSO@5.000 (5.000 cm)	68.9 (65.0 ± 2.6)	84.4 (81.2–87.2)	0.41 [0.30–0.55]	<0.001	<0.001	***
*Hynobius* *yangi*	DMF@10.00 (5.000 cm)	DMSO@15.00 (10.00 cm)	0.9 (0.2 ± 0.1)	10.9 (7.3–76.1)	0.08 [0.00–0.49]	<0.001	0.001	**
	DMSO@10.00 (5.000 cm)	DMSO@15.00 (10.00 cm)	26.1 (18.2 ± 5.9)	10.9 (7.3–76.1)	2.86 [1.42–5.80]	0.002	0.002	**
	DMF@5.000 (10.00 cm)	DMSO@15.00 (10.00 cm)	3.5 (1.5 ± 0.9)	10.9 (7.3–76.1)	0.30 [0.09–0.83]	0.013	0.018	*
	DMF@15.00 (5.000 cm)	DMSO@15.00 (10.00 cm)	19.7 (13.5 ± 6.7)	10.9 (7.3–76.1)	1.94 [0.99–3.82]	0.038	0.051	ns
	DMF@15.00 (10.00 cm)	DMSO@15.00 (10.00 cm)	6.2 (3.1 ± 2.3)	10.9 (7.3–76.1)	0.54 [0.19–1.39]	0.219	0.283	ns
	DMSO@10.00 (10.00 cm)	DMSO@15.00 (10.00 cm)	6.8 (3.0 ± 5.1)	10.9 (7.3–76.1)	0.60 [0.17–1.72]	0.366	0.435	ns
	DMSO@15.00 (5.000 cm)	DMSO@15.00 (10.00 cm)	14.5 (9.7 ± 1.1)	10.9 (7.3–76.1)	1.38 [0.68–2.78]	0.405	0.469	ns
	DMSO@5.000 (5.000 cm)	DMSO@15.00 (10.00 cm)	8.5 (5.3 ± 3.4)	10.9 (7.3–76.1)	0.76 [0.36–1.58]	0.49	0.539	ns
	DMF@5.000 (5.000 cm)	DMSO@15.00 (10.00 cm)	10.3 (6.3 ± 6.3)	10.9 (7.3–76.1)	0.93 [0.43–1.98]	1	1	ns
	DMF@10.00 (10.00 cm)	DMSO@15.00 (10.00 cm)	0.0 (0.0 ± 0.4)	10.9 (7.3–76.1)	0.00 [0.00–0.22]	<0.001	<0.001	***
	DMSO@5.000 (10.00 cm)	DMSO@15.00 (10.00 cm)	1.4 (0.4 ± 0.8)	10.9 (7.3–76.1)	0.11 [0.01–0.47]	<0.001	<0.001	***
*Kaloula* *borealis*	DMSO@5.000 (10.00 cm)	DMSO@5.000 (5.000 cm)	5.3 (3.8 ± 0.2)	5.1 (3.8–4.7)	1.04 [0.64–1.66]	0.909	0.976	ns
	DMF@10.00 (10.00 cm)	DMSO@5.000 (5.000 cm)	41.8 (37.2 ± 6.5)	5.1 (3.8–4.7)	13.37 [9.29–19.56]	<0.001	<0.001	***
	DMF@10.00 (5.000 cm)	DMSO@5.000 (5.000 cm)	42.4 (38.6 ± 6.4)	5.1 (3.8–4.7)	13.72 [9.70–19.75]	<0.001	<0.001	***
	DMF@15.00 (10.00 cm)	DMSO@5.000 (5.000 cm)	77.8 (74.3 ± 0.9)	5.1 (3.8–4.7)	64.97 [45.07–94.96]	<0.001	<0.001	***
	DMF@15.00 (5.000 cm)	DMSO@5.000 (5.000 cm)	69.6 (65.5 ± 3.5)	5.1 (3.8–4.7)	42.65 [29.68–62.15]	<0.001	<0.001	***
	DMF@5.000 (10.00 cm)	DMSO@5.000 (5.000 cm)	73.1 (69.9 ± 6.1)	5.1 (3.8–4.7)	50.49 [35.82–72.56]	<0.001	<0.001	***
	DMF@5.000 (5.000 cm)	DMSO@5.000 (5.000 cm)	40.0 (36.5 ± 3.5)	5.1 (3.8–4.7)	12.40 [8.84–17.71]	<0.001	<0.001	***
	DMSO@10.00 (10.00 cm)	DMSO@5.000 (5.000 cm)	50.0 (44.7 ± 5.3)	5.1 (3.8–4.7)	18.58 [12.73–27.57]	<0.001	<0.001	***
	DMSO@10.00 (5.000 cm)	DMSO@5.000 (5.000 cm)	81.6 (77.3 ± 5.5)	5.1 (3.8–4.7)	82.97 [54.60–128.41]	<0.001	<0.001	***
	DMSO@15.00 (10.00 cm)	DMSO@5.000 (5.000 cm)	61.5 (57.2 ± 5.6)	5.1 (3.8–4.7)	29.61 [20.76–43.08]	<0.001	<0.001	***
	DMSO@15.00 (5.000 cm)	DMSO@5.000 (5.000 cm)	62.0 (57.9 ± 5.9)	5.1 (3.8–4.7)	30.33 [21.37–43.89]	<0.001	<0.001	***
*Pelophylax chosenicus*	DMF@15.00 (10.00 cm)	DMF@5.000 (10.00 cm)	67.8 (64.5 ± 0.9)	74.4 (71.4–77.2)	0.72 [0.58–0.90]	0.003	0.004	**
	DMF@10.00 (10.00 cm)	DMF@5.000 (10.00 cm)	75.5 (73.1 ± 8.9)	74.4 (71.4–77.2)	1.10 [0.87–1.37]	0.433	0.488	ns
	DMSO@10.00 (5.000 cm)	DMF@5.000 (10.00 cm)	74.4 (71.3 ± 7.4)	74.4 (71.4–77.2)	1.00 [0.80–1.26]	1	1	ns
	DMF@10.00 (5.000 cm)	DMF@5.000 (10.00 cm)	28.1 (25.0 ± 1.5)	74.4 (71.4–77.2)	0.13 [0.11–0.17]	<0.001	<0.001	***
	DMF@15.00 (5.000 cm)	DMF@5.000 (10.00 cm)	41.8 (38.5 ± 5.1)	74.4 (71.4–77.2)	0.25 [0.20–0.30]	<0.001	<0.001	***
	DMF@5.000 (5.000 cm)	DMF@5.000 (10.00 cm)	38.0 (34.9 ± 1.3)	74.4 (71.4–77.2)	0.21 [0.17–0.26]	<0.001	<0.001	***
	DMSO@10.00 (10.00 cm)	DMF@5.000 (10.00 cm)	49.0 (45.6 ± 2.4)	74.4 (71.4–77.2)	0.33 [0.27–0.41]	<0.001	<0.001	***
	DMSO@15.00 (10.00 cm)	DMF@5.000 (10.00 cm)	55.3 (52.0 ± 8.6)	74.4 (71.4–77.2)	0.43 [0.35–0.52]	<0.001	<0.001	***
	DMSO@15.00 (5.000 cm)	DMF@5.000 (10.00 cm)	49.0 (45.4 ± 2.6)	74.4 (71.4–77.2)	0.33 [0.27–0.41]	<0.001	<0.001	***
	DMSO@5.000 (10.00 cm)	DMF@5.000 (10.00 cm)	16.6 (13.9 ± 9.6)	74.4 (71.4–77.2)	0.07 [0.05–0.09]	<0.001	<0.001	***
	DMSO@5.000 (5.000 cm)	DMF@5.000 (10.00 cm)	59.7 (56.3 ± 3.1)	74.4 (71.4–77.2)	0.51 [0.41–0.63]	<0.001	<0.001	***

Abbreviations: DMSO, dimethyl sulfoxide; DMF, N,N-dimethylformamide. Symbols: “@” denotes the final cryoprotectant concentration, and parentheses denote the pre-freezing height (cm). Proportions are shown as mean (95% CI) using Wilson intervals. Odds ratios are computed versus the species-specific baseline condition shown in the “Baseline” column. *p*-values are Benjamini–Hochberg FDR-adjusted. Significance codes: ns *p* ≥ 0.05, * *p* < 0.05, ** *p* < 0.01, *** *p* < 0.001.

**Table 4:** Model-based marginal predictions of post-thaw membrane-integrity viability (mean ± SE, %; 95% CI) across species and cryoprotectants (all with 0.6 M sucrose).

Species	Cryoprotectant	Predicted Integral Membrane (%)	95% CI
*Dryophytes suweonensis*	DMF	73.2% ± 0.6% [b]	71.9–74.4%
	DMSO	70.4% ± 0.7% [a]	69.1–71.7%
*Pelophylax chosenicus*	DMF	54.9% ± 0.6% [b]	53.7–56.0%
	DMSO	51.5% ± 0.6% [a]	50.2–52.7%
*Kaloula borealis*	DMF	50.2% ± 0.7% [b]	48.9–51.5%
	DMSO	46.8% ± 0.7% [a]	45.4–48.2%
*Hynobius yangi*	DMF	8.8% ± 0.7% [b]	7.5–10.3%
	DMSO	7.8% ± 0.6% [a]	6.6–9.1%

Sample sizes (post-thaw technical replicates, straws): *D. suweonensis n* = 84; *P. chosenicus n* = 84; *K. borealis n* = 60; *H. yangi n* = 60. Biological replicates (unique individuals): *D. suweonensis n* = 15, *P. chosenicus n* = 15, *K. borealis n* = 15, *H. yangi n* = 5. Error term = SE. Letters (a, b) denote BH–FDR-adjusted within-species pairwise differences (α = 0.05).

**Table 5:** Species-specific optimal cryopreservation conditions (mean ± SE, %; 95% CI).

Species	Condition	Cryoprotectant	Mean ± SE (%)	95% CI (%)	Total (Cells)	No. of Individuals	Straws Per Conditions
*Dryophytes suweonensis*	DMSO@15.00 (10.00 cm)	DMSO	86.5 ± 2.3 [g]	83.3–89.3	499	15	7
*Hynobius yangi*	DMF@15.00 (5.000 cm)	DMF	19.7 ± 5.2 [ef]	13.5–26.7	135	5	5
*Kaloula borealis*	DMSO@10.00 (5.000 cm)	DMSO	81.6 ± 2.3 [g]	77.3–85.5	345	15	5
*Pelophylax chosenicus*	DMF@10.00 (10.00 cm)	DMF	75.5 ± 2.6 [g]	73.1–78.9	825	15	7

Species-level technical replicates (post-thaw straws): *D. suweonensis N* = 84; *P. chosenicus N* = 84; *K. borealis N* = 60; *H. yangi N* = 60. Biological replicates (males): 15, 15, 15, and 5, respectively. Error term = SE across straws; 95% CIs are Wilson intervals computed from aggregated counts (alive/total). Letters (e, f, g) denote BH–FDR-adjusted within-species multiple-comparison groups (α = 0.05); conditions sharing ≥1 letter are not significantly different. “@” denotes the final cryoprotectant concentration; parentheses denote the pre-freezing height (cm). Abbreviations: DMSO, dimethyl sulfoxide; DMF, N,N-dimethylformamide.

## Data Availability

The data presented in this study are available on request from the corresponding author.
